# Analysis of miRNAs Involved in Mouse Brain Damage upon Enterovirus 71 Infection

**DOI:** 10.3389/fcimb.2017.00133

**Published:** 2017-04-19

**Authors:** Xiaoxia Yang, Jing Xie, Leili Jia, Nan Liu, Yuan Liang, Fuli Wu, Beibei Liang, Yongrui Li, Jinyan Wang, Chunyu Sheng, Hao Li, Hongbo Liu, Qiuxia Ma, Chaojie Yang, Xinying Du, Shaofu Qiu, Hongbin Song

**Affiliations:** ^1^Center for Infectious Disease Control, Institute of Disease Control and Prevention, Academy of Military Medical SciencesBeijing, China; ^2^The Key Laboratory of Pharmacology and Molecular Biology, Medical College, Henan University of Science and TechnologyLuoyang, China

**Keywords:** enterovirus 71 (EV71), miRNA, brain, central nervous system, inflammation, neural system, brainstem encephalitis

## Abstract

Enterovirus 71 (EV71) infects the central nervous system (CNS) and causes brainstem encephalitis in children. MiRNAs have been found to play various functions in EV71 infection in human cell lines. To identify potential miRNAs involved in the inflammatory injury in CNS, our study, for the first time, performed a miRNA microarray assay *in vivo* using EV71 infected mice brains. Twenty differentially expressed miRNAs were identified (four up- and 16 down-regulated) and confirmed by qRT-PCR. The target genes of these miRNAs were analyzed using KEGG (Kyoto Encyclopedia of Genes and Genomes) analysis, revealing that the miRNAs were mainly involved in the regulation of inflammation and neural system function. MiR-150-5p, -3082-5p, -3473a, -468-3p, -669n, -721, -709, and -5107-5p that regulate MAPK and chemokine signaling were all down-regulated, which might result in increased cytokine production. In addition, miR-3473a could also regulate focal adhesion and leukocyte trans-endothelial migration, suggesting a role in virus-induced blood-brain barrier disruption. The miRNAs and pathways identified in this study could help to understand the intricate interactions between EV71 and the brain injury, offering new insight for the future research of the molecular mechanism of EV71 induced brainstem encephalitis.

## Introduction

Enterovirus 71 (EV71) is one of the main pathogens responsible for hand, foot, and mouth disease (HFMD) in young children, generally causing a fever and rash. However, because of its neural invasive ability, the EV71 infection can also lead to the development of severe neurological symptoms, including aseptic meningitis, brainstem encephalitis, and flaccid paralysis (Ooi et al., 2010). For this reason, apart from poliovirus, EV71 is considered the most dangerous childhood pathogen (Ooi et al., 2010). Central nervous system (CNS) inflammation is thought to be the principal underlying cause of the pathogenesis caused by EV71 infection. In addition, complications arising from CNS inflammation, like pulmonary oedema and myocarditis are the main causes of death (Ye et al., 2015). Post-mortem histopathological examination of EV71 infected individuals has shown a high degree of inflammation, oedema, and necrosis in the brainstem and spinal cord, accompanied by lymphocyte and macrophage infiltration (Zhang et al., 2012). These observations indicate that inflammatory damage in the brain plays a crucial role in the pathogenesis of EV71 infection. Therefore, understanding the inflammatory pathogenesis of EV71 infection in the brain is essential for the prevention, control, and future treatment.

MicroRNAs (miRNAs) are endogenous non-coding RNAs, ~20–22 nucleotides long, that bind to a complementary sequence located in the 3′UTR of their target mRNA, leading to the formation of the RNA-induced silencing complex (RISC), which then either prevents mRNA translation or triggers its degradation, therefore, controlling gene expression by post-transcriptional regulation (Xiao and Rajewsky, 2009). Growing evidence suggests that miRNAs play an important role in host-virus interactions by either positively or negatively regulating viral infection, thereby affecting host pathology. For example, human miR-32 could inhibit the replication of primate foamy virus type 1 (PFV-1) (Lecellier et al., 2005), miR-122 was found to be up-regulated following severe liver damage and inflammation in chronic hepatitis C infection (Gholami et al., 2016), and mice depleted of miR-155 were significantly more susceptible to herpes simplex induced encephalitis (Bhela et al., 2014), implying that there is a regulatory role for miRNA in the inflammatory damage caused by virus infection.

The expression of miRNAs had been reported to be altered during EV71 infection *in vitro*; miR-526a and miR-146a that target the RIG-I-dependent innate immune response and the IRAK1 pathway respectively, were both up-regulated upon EV71 infection, thus inhibiting type I interferon production, facilitating further EV71 infection (Ho et al., 2011; Xu et al., 2014). However, the CNS possesses a unique blood-brain barrier (BBB) that serves to protect the CNS from invasion by pathogens, inflammatory cells, or other deleterious molecules (Spindler and Hsu, 2012). Vascular dilatation and infiltration of inflammatory cells such as lymphocytes and macrophages that have been observed in the brainstem of EV71-infected patients (Zhang et al., 2012) likely arose because of a complicated interaction between the virus, lymphocytes/macrophages, the BBB, and the host immune system. To date, all the research examining EV71 related miRNAs have been performed using human cell lines. These studies were limited however because they did not describe the integral mechanism underlying the pathology. Therefore, in order to identify potential miRNAs and understand the roles they might play in EV71 induced inflammatory damage, we exploited the EV71-infected suckling mouse model to study miRNA expression profiles in mouse brain.

## Materials and methods

### Virus

The EV71 virus used in this study was strain AH08/06 (accession number HQ611148 in the GenBank) isolated in 2010 from Anhui province, China. The virus was propagated in RD (rhabdomyosarcoma) cells and virus titer was determined by TCID_50_ (Liu et al., 2016).

### EV71 infection in mice and ethics statement

One-day-old suckling Kunming (KM) mice were obtained from the Laboratory Animal Centre of the Academy of Military Medical Sciences (AMMS), China. The mice were injected intraperitoneally with 100 μL virus suspension (10^9^ TCID_50_) or DMEM cell culture medium (*n* = 10/group). Disease manifestation and mouse weight were monitored daily post-infection. The disease symptoms of mice were scored as follow: 0: healthy; 1: reduced mobility; 2: wasting; 3: limb weakness; 4: limb paralysis; and 5: moribund or death. Mouse brain were harvested, weighed, homogenized, and EV71 titers were detected in RD cells at 3 and 5 days post-infection (dpi) (*n* = 3/day) (Reddy et al., 2011). Inflammatory changes in mouse brain were observed by haematoxylin-eosin (HE) staining (Lin et al., 2015).

All animal procedures were conducted under protocols approved by the Institute of Animal Care and Use Committee at AMMS.

### MiRNA isolation

For miRNA extraction, entire brains from mice infected by EV71 or negative controls (*n* = 3/group) were harvested on days 3 and 5 post-infection respectively. The brain tissues were homogenized in PBS using a high-throughput organization grinding apparatus (Scientz-48, Scientz, Ningbo, China). MiRNA was extracted from homogenized brains using the miRNA Purification Kit (Cowin Biotech, Beijing, China) according to the manufacturer's protocol.

### MiRNA microarray analysis

Microarray analysis of the isolated miRNA was performed by CapitalBio Corporation (Beijing, China). Agilent mouse miRNA microarray slides (Release V18.0) (Agilent Technologies, Santa Clara, CA, USA) were used according to the manufacturer's instructions. Briefly, 200 ng of miRNA sample was dephosphorylated at the 5′ end using alkaline phosphatase (CIP) provided in the miRNA Complete Labeling and Hyb Kit (Agilent Technologies, Santa Clara, CA, USA) and the 3′ end-linked with a Cyanine3-pCp using T4 RNA ligase. The concentrated and dried reaction mixtures were hybridized with the Agilent microarray overnight in an Agilent hybridization oven (Agilent Technologies, Santa Clara, CA, USA). The chip slides were then washed with a 2 × saline sodium citrate (SSC) wash buffer containing 0.2% SDS at 42°C for 5 min and 0.2 × SSC for 5 min. The chips were then scanned on an Agilent Microarray Scanner (Agilent, Model #G2565CA) and results were extracted utilizing Agilent Feature Extraction software (v10.7, Agilent). The data was normalized and differential gene expression analyzed using GeneSpring software (Agilent). MiRNAs with a relative expression level normalized to the control that showed a fold change (FC) ≥2 and a *P* value ≤0.05 were considered to be significantly up-regulated, and those with FC ≤ −2 and a *P* value ≤0.05 were considered to be significantly down-regulated.

### Real-time PCR

To validate the miRNA microarray results, we used Real-time PCR to detect the miRNA expression in mice brain (*n* = 4~5/group). For cDNA synthesis, we used the polyA tailing method using miRNA cDNA Synthesis Kit (Cowin Biotech, Beijing, China) according to the supporting protocol. Briefly, a polyA tail was added to the miRNA by incubating *E. coli* poly(A) Polymerase supplied with the kit with the miRNA at 37°C for 15 min, followed by incubation at 42°C for 50 min with RT primers [with an oligo (dT) universal tag] and super reverse transcriptase (RT). Real-time PCR was performed using a miRNA qPCR Assay Kit (Cowin Biotech, Beijing, China). The reverse primer used in this step was the universal adaptor PCR primer provided with the assay kit and the forward primers used are listed in Supporting File 1. A two-step cycling program was performed for 45 cycles: 95°C for 10 s, 58°C for 60 s. U6 was used as reference gene for normalization and differential gene expression data was obtained using the 2^−ΔΔCt^ method (Livak and Schmittgen, 2001). The statistical power of each miRNA was calculated using GPower 3.1 and the power was both greater than 0.9.

### Target prediction of differentially expressed miRNAs

Potential miRNA targets were predicted using predicted microRNA-gene target module of miRWalk 2.0 (version 15.02.2016, http://zmf.umm.uni-heidelberg.de/apps/zmf/mirwalk2/miRretsys-self.html) under the programs of miRWalk, miRanda and Targetscan together. The predicted targets underwent KEGG pathway analysis using the functional annotation tools of DAVID 6.8 (Beta) (https://david-d.ncifcrf.gov/tools.jsp), using the set of mouse genes as a background set. The enrichment of KEGG pathways was identified with a cut-off standard of *P* < 0.01. KEGG pathway analysis for each miRNA was conducted using the miRNA-gene targets tool of miRWalk 2.0 using a cut-off of *P* < 0.05 (Benjamini-Hochberg false discovery rate, FDR correction) (Liu et al., 2015). KEGG pathways identified by miRWalk went though further validation by DAVID v6.8 and pathways that with *P* < 0.01 and gene number> 30 were considered credible.

### Microarray data resource

The microarray data were deposited in the Gene Expression Omnibus database (http://www.ncbi.nlm.nih.gov/geo/) under the accession number GSE89070.

## Results

### Establishment of the EV71 infected mouse model

**Figure 1:**
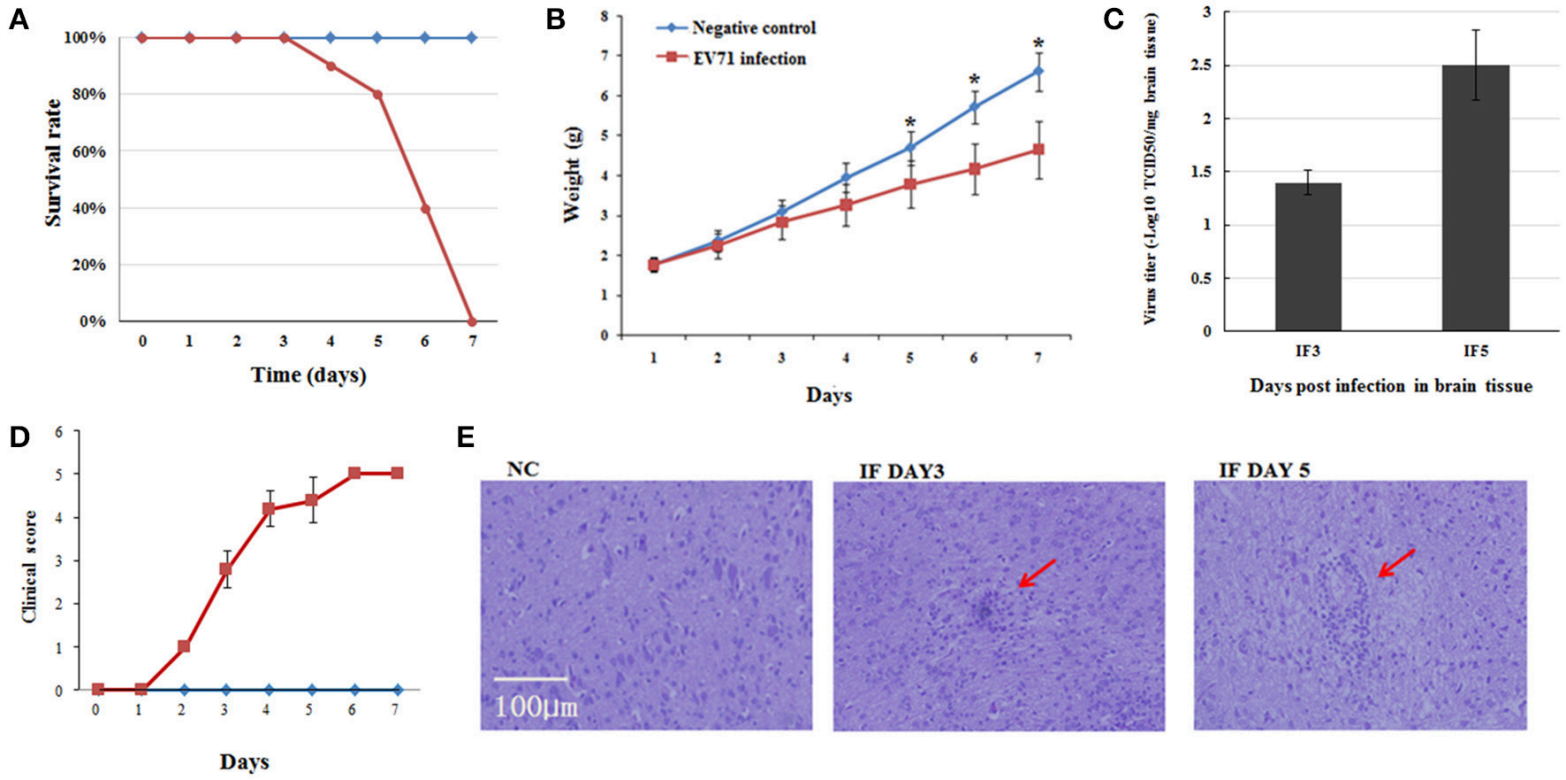
**Pathogenesis of EV71 infection in mice. (A)** Survival rates of EV71 infected mice vs. negative control mice. **(B)** Weight gain in EV71 infected mice. Mice were monitored for 7 days. Data are presented as mean ± standard deviation (*SD*), *n* = 3, ^*^P < 0.05. **(C)** Virus titer in EV71 infected mice brain, *n* = 3. **(D)** Clinical symptoms of EV71 infected mice, *n* = 10, data are presented as mean ± *SD*. **(E)** HE staining of mouse brain sections. NC, IF DAY3, and IF DAY5 represent uninfected negative control, and EV71 infected at 3 dpi and 5 dpi, respectively. The red arrows indicate monocyte infiltration.

We first set out to establish the EV71 infected mouse model using 1-day-old suckling mice. After intraperitoneal injection of EV71, the mice showed typical symptoms of infection, such as torpor and limb paralysis at 3 dpi. Mice began to die from the fourth day onward and almost all the mice had succumbed to EV71 infection at 7 dpi (Figure 1A). We also monitored weight gain and clinical symptoms over time and compared with the normal group. A lower rate of weight gain and severe clinical symptoms became evident in the infected group from 2 dpi and continued until their death (Figures 1B,D). The infection of EV71 in mouse brain was verified by detecting virus titer using TCID_50_ at 3 and 5 dpi (Figure 1C). Haematoxylin-eosin (HE) staining of EV71 infected mice brains revealed monocyte infiltration at 3 and 5 dpi (Figure 1E). These results suggested that we had successfully established the EV71 infected mouse model, which had inflammatory injury in brain.

### Regulation of miRNA profile in mouse brain in response to EV71 infection

**Figure 2:**
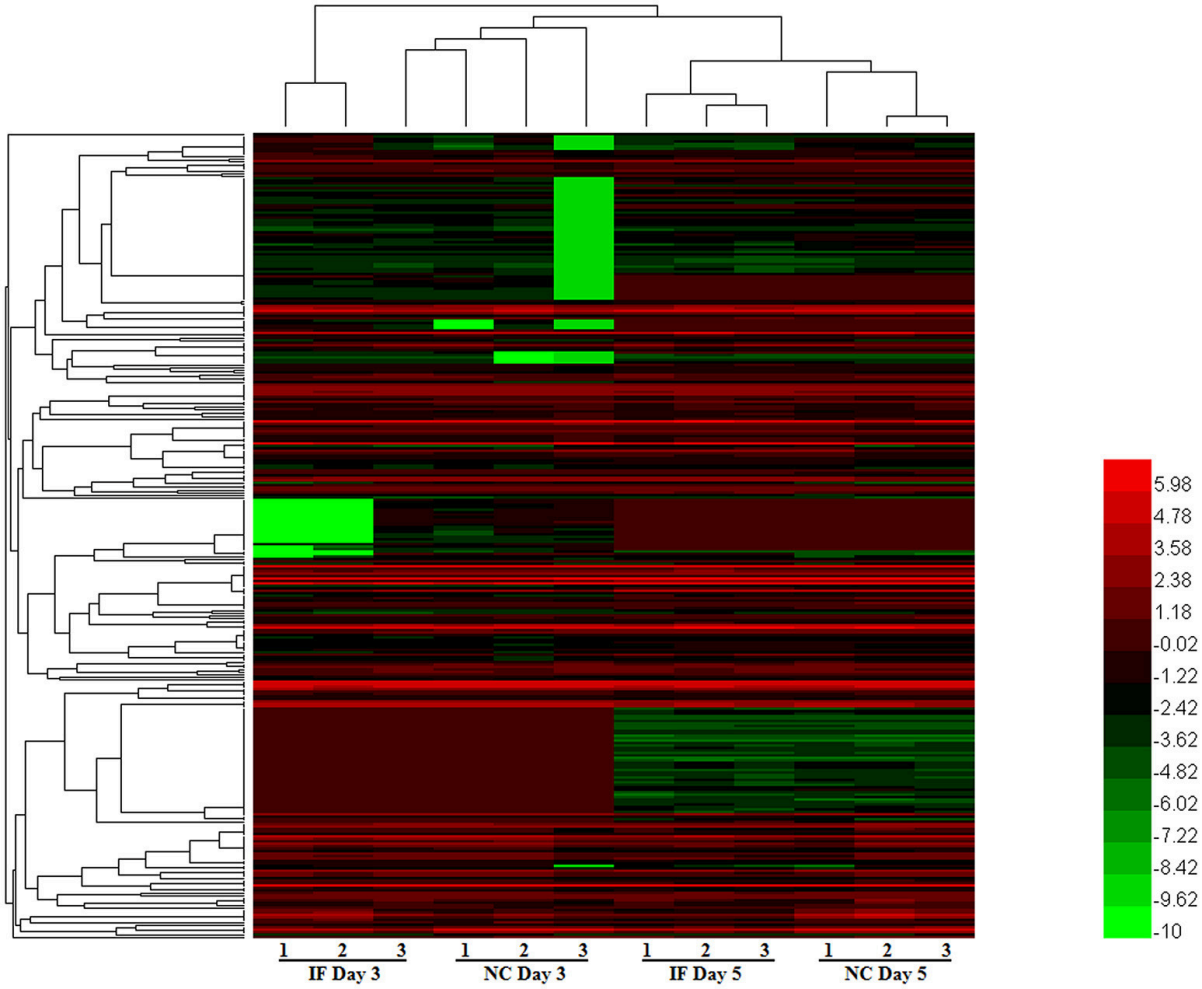
**The miRNA expression patterns in EV71 infected mice brains**. A total of 328 miRNAs were detected in EV71 infected mouse brain at 3 and 5 days post-infection. Their expression profiles are depicted using a clustered heatmap. The rows and columns correspond to different miRNAs and mice respectively. The different colors indicate different expression levels, red indicates up-regulation and green indicates down-regulation.

To evaluate miRNA expression changes caused by EV71 infection in mouse brain, we determined the miRNA expression profiles at 3 and 5 days post-infection. Based on the microarray analysis, there were 328 miRNAs detected in mouse brain. The expression patterns of these miRNAs are displayed in the clustered heatmap shown in Figure 2. The expression profile induced by EV71 infection at day 3 was quite distinct from the profile seen at day 5. In the latter case, a larger number of miRNAs was observed. At day 3 post-infection, there were only two down-regulated miRNAs, miRNA-3473a and miRNA-3473b, and no up-regulated miRNAs. At day 5 post-infection, there were four up-regulated and 16 down regulated miRNAs respectively, as shown in Table 1. Importantly, the two miRNAs, namely miRNA-3473a and miRNA-3473b, which were down-regulated at 3 days post-infection, were also down-regulated at 5 days post-infection to almost the same extent.

**Table 1:** **Expression profiles of differentially expressed miRNAs**.

**miRNA**	**Fold change at 3 dpi**	**Fold change at 5 dpi**
mmu-miR-466h-3p	NS (Not significant)	14.311053
mmu-miR-346-5p	NS	3.4766614
mmu-miR-877-3p	NS	3.416667
mmu-miR-7a-5p	NS	2.1413074
mmu-miR-5107-5p	NS	−2.047792
mmu-miR-3473a	−2.2872427	−2.1317267
mmu-miR-150-5p	NS	−2.1770155
mmu-miR-3473b	−3.2475147	−2.282881
mmu-miR-721	NS	−2.6864858
mmu-miR-669b-5p	NS	−2.9408455
mmu-miR-709	NS	−3.0065749
mmu-miR-669n	NS	−3.0094464
mmu-miR-468-3p	NS	−3.40051
mmu-miR-466m-5p	NS	−4.33538
mmu-miR-32-3p	NS	−4.5324426
mmu-miR-466h-5p	NS	−4.9673104
mmu-miR-3082-5p	NS	−6.01648
mmu-miR-466i-5p	NS	−7.6776285
mmu-miR-1187	NS	−8.772696
mmu-miR-574-5p	NS	−9.259378

### Verification of differentially expressed miRNAs by real-time PCR

**Figure 3:**
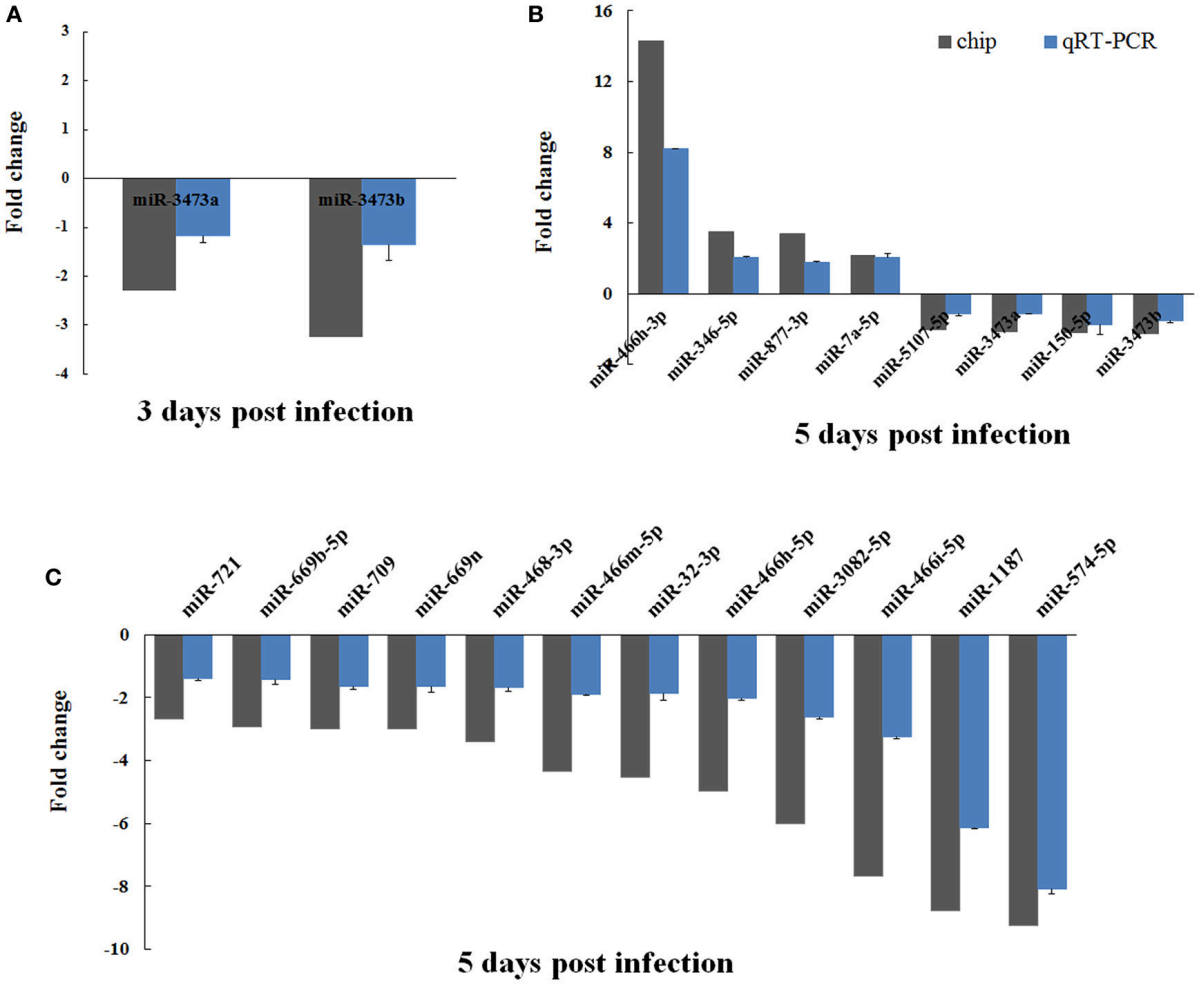
**Verification of microarray data using real-time PCR**. The expression of the 20 differentially expressed miRNAs identified by microarray analysis were analyzed using real-time PCR. The two miRNAs changed by 3 days post-infection are shown in **(A)**. The 20 miRNAs changed at 5 days post-infection are shown in **(B)** and **(C)**. The detection for each miRNA was repeated three times and the standard deviation was denoted as error bar.

To confirm the validity of the differentially expressed miRNAs that had been identified by microarray analysis, we performed real-time PCR on all 20 of these miRNAs using the polyA tailing technique. The fold changes in expression of these miRNAs in EV71 infected mice brain were calculated, using uninfected mice brain for normalization. Overall, the real-time PCR data agreed well with the microarray analysis in terms of identifying up- and down-regulated miRNAs. However, the absolute magnitude of the changes differed between the two (Figure 3). In general, the fold changes detected by real-time PCR were lower than those found in the microarray analysis. This difference might be caused by the higher sensitivity of microarray detection compared to qRT-PCR.

### Targets prediction and pathway analysis of differentially expressed miRNAs

To understand the roles tha t the differentially expressed miRNAs played in response to EV71 infection, we predicted the potential targets regulated by these miRNAs using the miRNA-gene target module of miRWalk 2.0 (version 15.02.2016) adopting an approach utilizing the miRWalk database, the miRanda algorithm, and Targetscan software together. After de-duplication, 12,923 target genes were obtained (see Supporting File 2).

**Figure 4:**
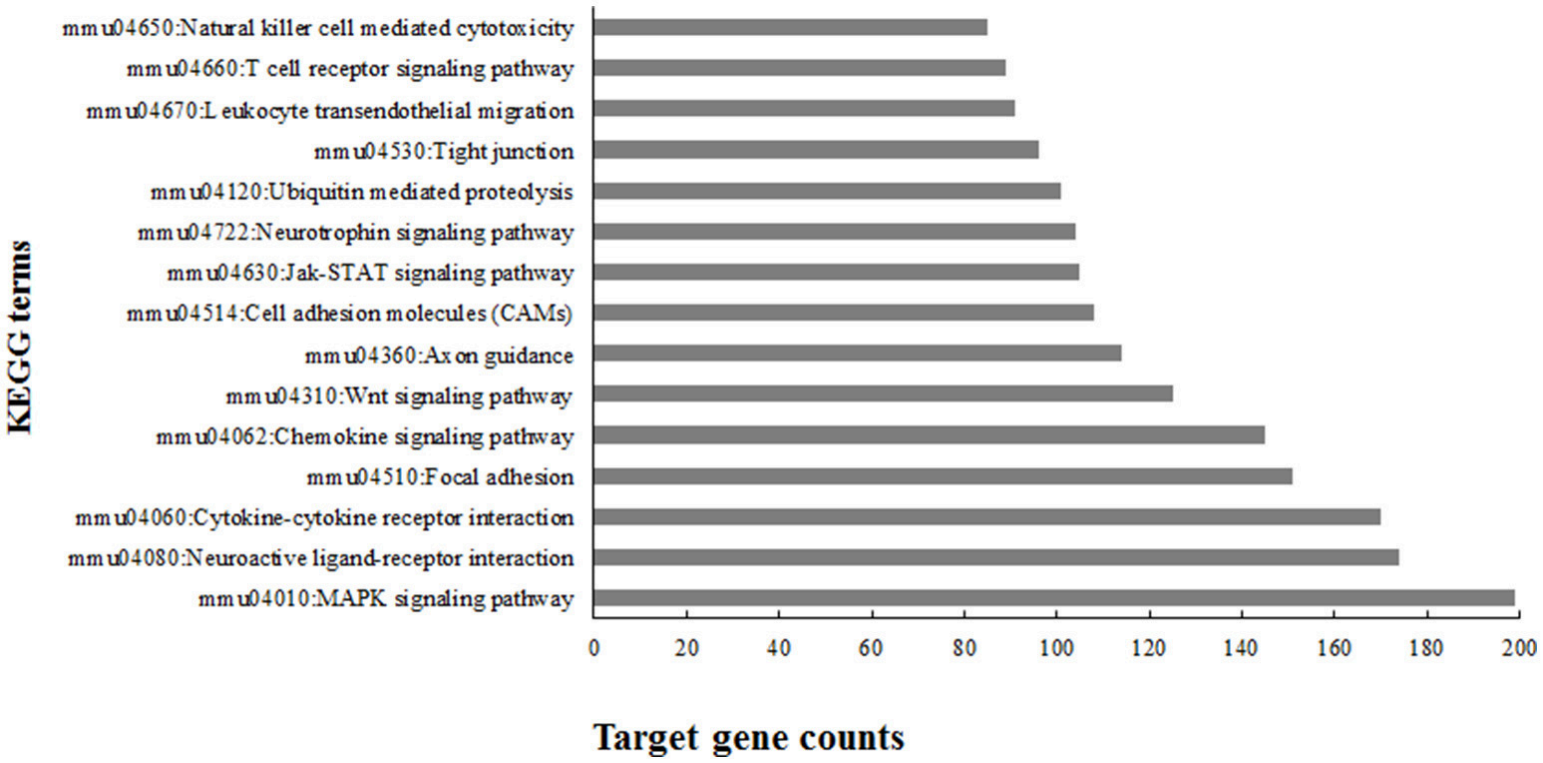
**Top 15 enriched KEGG terms using predicted targets of differentially expressed microRNAs**.

To gain an understanding of the overall role these miRNAs played in EV71 infection, KEGG (Kyoto Encyclopedia of Genes and Genomes) pathway analysis was performed using the 12,923 target genes in the database DAVID v6.8. The KEGG pathway terms were ranked according to the target gene counts (*p* < 0.01) and the top 15 terms associated with EV71 infected inflammatory damage are listed in Figure 4. The pathways were mainly involved in anti-viral immunity, cytokine production, neural signaling, and maintenance of BBB structure, suggesting the important roles these miRNAs might play in EV71 infection.

To elucidate the corresponding miRNAs that played a role in the different processes involved in EV71 infection, the KEGG pathways regulated by each miRNA respectively were analyzed using miRWalk 2.0 (FDR < 0.05; Liu et al., 2015). Target genes for each miRNA were put into DAVID v6.8 (*p* < 0.01) for simultaneous analysis to identify genes involved in each KEGG pathway. There were 10 miRNAs identified with the KEGG terms (gene number > 30) (see Supporting File 3) associated with EV71 infection. The KEGG terms mainly referred to the neural system and inflammatory response, containing pathways involved in axonal guidance, focal adhesion, MAPK signaling, and chemokine signaling pathways (Tables 2, 3). Most of the identified miRNAs, including miR-150-5p, -3082-5p, -3473a, -468-3p, -5107-5p, -669n, and -721 were mainly involved in the MAPK signaling pathway. MiR-709 could regulate the chemokine signaling pathway and miR-3473a might regulate leukocyte trans-endothelial migration. In addition, miR-3473a and 3473b could involve in axon guidance and the Wnt signaling pathway. Intriguingly, the analysis revealed that the four up-regulated miRNAs showed little participation in the two processes. All of the identified miRNAs were down-regulated upon EV71 infection.

**Table 2:** **The changed miRNAs that involved in nervous system upon EV71 infection**.

**miRNA**	**Pathway name**	**BH value**	**Gene counts**
mmu-miR-1187	Wnt signaling pathway	0.0048	34
mmu-miR-1187	Axon guidance	0.0097	30
mmu-miR-1187	Neuroactive ligand receptor interaction	0.0235	49
mmu-miR-3082-5p	Axon guidance	0.0000	39
mmu-miR-3082-5p	Focal adhesion	0.0002	46
mmu-miR-3473a	ErbB signaling pathway	0.0000	33
mmu-miR-3473a	Focal adhesion	0.0000	55
mmu-miR-3473a	Wnt signaling pathway	0.0000	44
mmu-miR-3473a	Axon guidance	0.0000	39
mmu-miR-3473a	Cell adhesion molecules CAMs	0.0188	36
mmu-miR-3473a	Leukocyte transendothelial migration	0.0256	30
mmu-miR-3473b	Axon guidance	0.0000	36
mmu-miR-3473b	Wnt signaling pathway	0.0023	33
mmu-miR-5107-5p	Focal adhesion	0.0063	42
mmu-miR-5107-5p	Axon guidance	0.0070	31
mmu-miR-669n	Cell adhesion molecules CAMs	0.0004	36
mmu-miR-669n	Neuroactive ligand receptor interaction	0.0051	52
mmu-miR-669n	Wnt signaling pathway	0.0072	34
mmu-miR-709	Wnt signaling pathway	0.0001	51
mmu-miR-709	Neurotrophin signaling pathway	0.0011	43
mmu-miR-709	ErbB signaling pathway	0.0064	31
mmu-miR-709	Axon guidance	0.0136	40
mmu-miR-709	Focal adhesion	0.0282	54

**Table 3:** **The changed miRNAs that involved in inflammatory response upon EV71 infection**.

**miRNA**	**Pathway name**	**FDR value**	**Gene counts**
mmu-miR-150-5p	MAPK signaling pathway	0.0063	49
mmu-miR-3082-5p	Chemokine signaling pathway	0.0006	43
mmu-miR-3082-5p	MAPK signaling pathway	0.0015	54
mmu-miR-3473a	MAPK signaling pathway	0.0107	56
mmu-miR-468-3p	MAPK signaling pathway	0.0003	40
mmu-miR-5107-5p	MAPK signaling pathway	0.0118	51
mmu-miR-669n	MAPK signaling pathway	0.0021	54
mmu-miR-709	Chemokine signaling pathway	0.0094	53
mmu-miR-721	MAPK signaling pathway	0.0468	35

## Discussion

Emerging evidence had shown the important function miRNAs played in anti-viral immunity and there were numerous reports of miRNAs that are connected to EV71 infection. To elucidate the molecular mechanism of immunologic injury in CNS caused by EV71 infection, our report, for the first time, utilized EV71 infected suckling mice brain for miRNA microarray analysis to reveal the miRNAs that worked on mice brain damage.

In response to EV71 infection, we identified 20 miRNAs at 5 dpi compared with only two at 3 dpi. This difference might reflect the severity of infection, as can be seen in Figure 1, at 5 dpi, the mortality and physical condition of the mice were significantly severe than at 3 dpi. KEGG analysis of the 20 differentially expressed miRNAs suggested that these miRNAs might play important roles during EV71 infection (Figure 4). The top ranking terms “MAPK signaling pathway” and “Cytokine-cytokine receptor interaction” suggested that the differentially expressed miRNAs were mainly involved in the inflammatory process. Other terms, like “Neuroactive ligand-receptor interaction” and “Neurotrophin signaling pathway” implied that these miRNAs could also mediate the function of nervous system. These data suggest that the miRNAs we identified are involved in a variety of processes following EV71 infection and work together as a network to result in fatal damage to the EV71 infected brain. Therefore, in order to understand these processes, it was necessary to identify the miRNAs that functioned within each pathway and the probable target genes associated with them.

**Figure 5:**
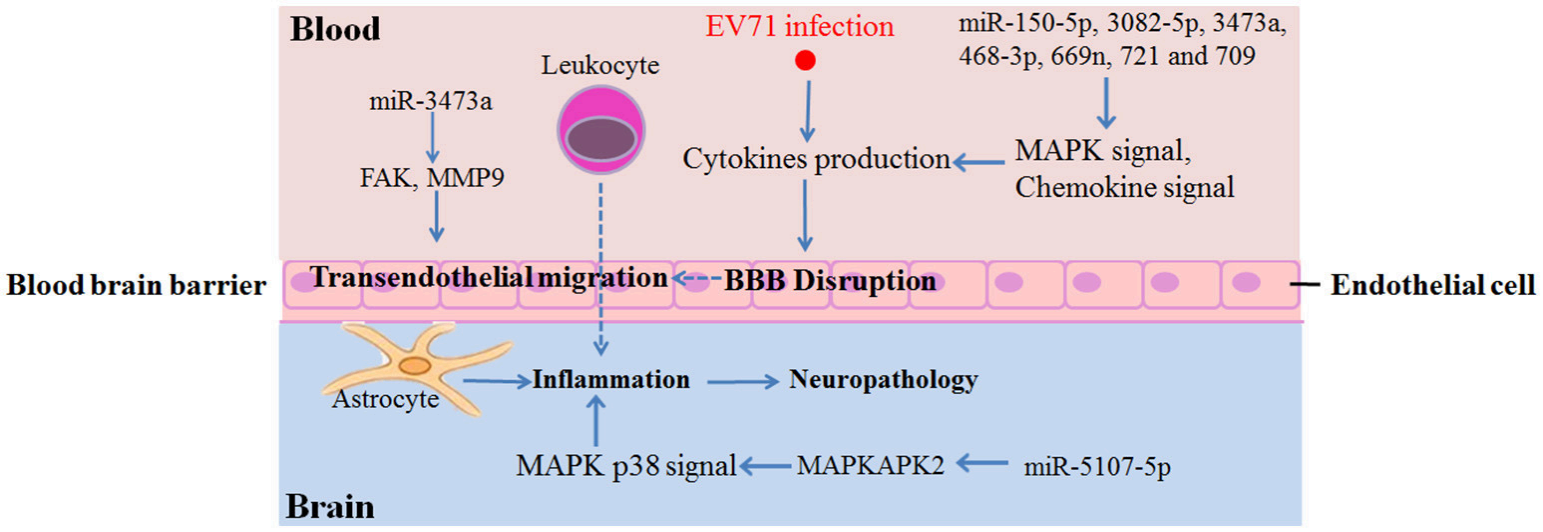
**Summary of identified miRNAs and the potential target genes involved in inflammatory damage in brain**.

Target gene analysis of each microRNA respectively by DAVID identified 10 miRNAs functioned upon EV71 infection. Interestingly, all 10 of the miRNAs were down-regulated upon EV71 infections, while the four up-regulated miRNAs showed no KEGG pathway enrichment (FDR < 0.05). We found that the majority of the identified miRNAs, including miR-150-5p, -3082-5p, -3473a, -468-3p, -5107-5p, -669n, and -721, were principally involved in MAPK signaling pathway that associated with inflammatory cytokines production (Kaminska et al., 2009). Among them, miR-5107-5p could regulate the MAPK p38 signaling pathway via the activation of MAPKAPK2 (MAPK activated protein kinase 2/MK2) in particularly (Figure 5 and Supporting File 3), thereby promoting the production of microglial-derived cytokines like TNF-α, IL-6, or IFN-γ, inside the BBB (Kaminska et al., 2009; Wang et al., 2015). Besides, another miRNA, miR-709, could regulate the chemokine signaling pathway by targeting genes belonging to the CCL and CXCL families.

Considering the negative regulation of target genes by miRNA, the down-regulation of these miRNAs could, as a result, activate these inflammation related pathways and promote inflammatory gene expression during EV71 infection. The fact that so many miRNAs are involved in the inflammatory response suggests that cytokine production in the brain is a complicated process and is controlled by a large molecular network. However, the important molecular players in this network have not yet been identified. Thus, it could help to pinpoint the key molecules in EV71-induced brain inflammation by further analyzing the potential targets of these miRNAs.

An interesting finding in our study was that there were only two miRNAs identified at 3 dpi, miR-3473a and -3473b, and they were both down-regulated. KEGG analysis revealed that they were both involved in axon guidance and the Wnt signaling pathway. Axon guidance directs an axon toward specific regions in the brain and Wnt signaling regulates neuronal positioning and axon development (Salinas and Zou, 2008; Van Battum et al., 2015). Considering that retrograde axonal transport is a major transmission route of EV71 (Chen et al., 2007), the down-regulation of miR-3473a and -3473b at an early stage of EV71 infection could invoke these neural pathways, thereby mediating EV71 transmission into the mouse brain. In addition, miR-3473a could also regulate focal adhesion and leukocyte trans-endothelial migration by targeting focal adhesion kinase (FAK/PTK2) and matrix metalloproteinase 9 (MMP9) genes respectively (Supporting File 3). Excessive expression of FAK in endothelial cells would be expected to weaken the continuity of the endothelial cellular barrier of BBB (Reddy et al., 2000; Arnold et al., 2013). MMPs are key factors that control BBB permeability by regulating the turnover and reconstruction of the extracellular matrix and MMP9 is the leading candidate protein implicated in accelerating BBB disruption (Savarin et al., 2011). Therefore, the down-regulation of miR-3473a would lead to over-expression of FAK and MMP9, resulting in BBB breaking. Inflammatory cell infiltration into the brainstem has been observed in a fatal EV71 infection, suggesting that BBB disruption had occurred (Yu et al., 2015). These results indicated that miR-3473a might play important roles in immunologic injury in the CNS upon EV71 infection.

The above analyses revealed that the differentially expressed miRNAs found upon EV71 infection in mouse brain were primarily associated with virus transmission, the inflammatory response and BBB disruption. Previously constructed miRNA profiles, using EV71 infected human cell lines, had mainly identified pathways that associated with antiviral immunity and inflammation (Bian et al., 2015; Xun et al., 2015). These profiles could hardly identify miRNAs involved in retrograde transmission and BBB disruption. In addition, the miRNAs involved were completely different from those identified in this study, suggesting that the miRNAs varied between different tissues and that a cell line could not represent the whole EV71 *in vivo* infection process. A report concerning prion infection in the CNS in a mouse model happened to support this conclusion. This report identified a number of miRNAs, including those identified in this study, namely miRNA-3473a, -3473b, and 709, which had the exact opposite expression profiles (Gao et al., 2016). These miRNAs were significantly up-regulated after prion infection, but were down-regulated after EV71 infection. Prion diseases are characterized by a failure to induce an inflammatory response, which is the exact opposite of the excessive inflammation seen upon EV71 infection. Thus, the opposite expression trend of miRNA-3473a, -3473b, and 709 strongly suggest that these miRNAs do have function on CNS inflammation. These results demonstrate that the mouse model is a better way to identify miRNAs involved in brain injury than cell lines. In addition, pathological lesions in EV71 infected mouse model are highly similar to the pathological changes seen in the CNS of human patient, including myelitis of the spinal cord, mononuclear cell infiltration and encephalitis in the brainstem (Yu et al., 2014). For the reasons described above, it is therefore appropriate to utilize a mouse model to study the molecular mechanism of inflammatory pathogenesis in the brain following EV71 infection. Thus, the miRNAs and probable targets and pathways identified in our work that relate to the inflammatory response and BBB disruption, could provide new insights into the molecular mechanism of EV71-mediated CNS inflammation and offer valuable targets for future research into this brainstem encephalitis disease. As a word of caution, however, the results reported here are based on miRNA target gene prediction and pathway analysis, more investigation will be required to verify the results in order to find efficient treatment strategies for EV71 mediated brainstem encephalitis.

## Author contributions

XY, JX, LJ, SQ, and HS designed the study. JX, LJ, and NL performed the animal experiment. BL, FW, YL, JW, CS, HL and HBL made the microarray analysis. XY, JX, and LJ carried out the pathway analysis. QM, YL, and XD verified the microarray results. XY, JX, LJ, SQ, and HS wrote the first draft of the manuscript. All authors reviewed the manuscript.

## Conflict of interest statement

The authors declare that the research was conducted in the absence of any commercial or financial relationships that could be construed as a potential conflict of interest.
