# Supplementary material for: Analysis of miRNAs Involved in Mouse Brain Damage upon Enterovirus 71 Infection
Source: Front Cell Infect Microbiol. 2017 Apr 19;7:133. doi: 10.3389/fcimb.2017.00133 (PMC5395563; doi:10.3389/fcimb.2017.00133)
Supplement: Supplementary file 1 [file Table1.DOCX]

Table 1. Forward primers for miRNA Real-Time PCR.

| **miRNA** | **Forward primer (5'-3')** |
| --- | --- |
| mmu-miR-466h-3p | TACGCACGCACACACACAC |
| mmu-miR-346-5p | TGTCTGCCCGAGTGCCTG |
| mmu-miR-877-3p | TGTCCTCTTCTCCCTCCTCCC |
| mmu-miR-7a-5p | GGGTGGAAGACTAGTGATTTTGTTGT |
| mmu-miR-5107-5p | TGGGCAGAGGAGGCAGGA |
| mmu-miR-3473a | GTGGAGAGATGGCTCAGCAAA |
| mmu-miR-150-5p | TCTCCCAACCCTTGTACCAGTG |
| mmu-miR-3473b | GGGCTGGAGAGATGGCTCAG |
| mmu-miR-721 | CAGTGCAATTAAAAGGGGGAAA |
| mmu-miR-669b-5p | GGAGTTTTGTGTGCATGTGCATG |
| mmu-miR-709 | GGAGGCAGAGGCAGGAGG |
| mmu-miR-669n | GGGATTTGTGTGTGGATGTGTGT |
| mmu-miR-468-3p | TATGACTGATGTGCGTGTGTCTG |
| mmu-miR-466m-5p | TGTGTGCATGTGCATGTGTG |
| mmu-miR-32-3p | GCCCCAATTTAGTGTGTGTGATATTA |
| mmu-miR-466h-5p | TGTGTGCATGTGCTTGTGTG |
| mmu-miR-3082-5p | GACAGAGTGTGTGTGTCTGTGT |
| mmu-miR-466i-5p | TGTGTGTGTGTGTGTGTGTG |
| mmu-miR-1187 | GGGGTATGTGTGTGTGTATGTGTGTAA |
| mmu-miR-574-5p | TGAGTGTGTGTGTGTGAGTGTG |
